# Elevated Thyroid Autoantibodies Aggravate Stroke Severity in Euthyroidism with Acute Ischemic Stroke

**DOI:** 10.1155/2022/8741058

**Published:** 2022-02-26

**Authors:** Jingyi Li, Shoulong Hu, Fang Liu, Dapeng Wu, Wei Song, Miao Hui

**Affiliations:** ^1^The Department of Endocrinology, Beijing United Family Hospital, Beijing, China; ^2^The Department of Ophthalmology, Beijing Children's Hospital, Capital Medical University, Beijing, China; ^3^The Department of Neurology, The First Affiliated Hospital, Tsinghua University, Beijing, China

## Abstract

**Introduction:**

Studies have indicated that immune reactions contribute to endothelial dysfunction and atherosclerosis. It is unclear whether thyroid dysfunction or elevated thyroid autoantibodies are associated with atherosclerosis. Therefore, we investigated the influence of thyroid autoimmunity related to elevated thyroid autoantibodies on functional outcome in euthyroidism with acute ischemic stroke (AIS).

**Methods:**

All patients with AIS underwent tests for thyroid function and thyroid antibodies (thyroid peroxidase antibody and thyroglobulin autoantibody). We divided the patients suffering from euthyroidism and AIS into positive thyroid autoantibody and negative thyroid autoantibody groups. Demographic profiles, risk factors, and functional outcomes were compared between the two groups.

**Results:**

Out of the total 422 patients, 50 (11.8%) were included in the positive thyroid autoantibody group. The National Institutes of Health Stroke Scale (NIHSS) score at admission and discharge was higher in the positive thyroid autoantibody group than the negative thyroid autoantibody group (*P* < 0.05). In addition, there was significant difference in the mortality during hospitalizations between the two groups (*P* < 0.01).

**Conclusion:**

This study showed that thyroid autoantibodies aggravate stroke severity in euthyroidism with AIS. We speculate that vascular damage related to thyroid autoimmunity may aggravate the increased risk of unfavorable outcomes, independent of thyroid function.

## 1. Introduction

Ischemic stroke is one of the leading causes of morbidity and mortality worldwide, and endothelial dysfunction and arteriosclerosis are its primary etiologies. Several factors such as age, smoking, obesity, hypertension, diabetes, and dyslipidemia have been recognized as aggravating the progress of arteriosclerosis. In addition, in recent years, some studies have highlighted that the immune responses are profoundly involved in the progression of endothelial dysfunction and arteriosclerosis [1]. Several cases have provided evidence for the association between thyroid diseases and arterial damage [2, 3]. A study showed the correlation between hyperthyroidism and intracranial arterial stenosis in stroke patients [4]. In addition, thyroid dysfunction and positive thyroid autoantibodies were shown in pediatric patients with moyamoya disease [5]. It is unclear whether thyroid dysfunction or positive thyroid autoantibodies were associated with vascular diseases. Therefore, we retrospectively analyzed our AIS patients with euthyroidism and compared the stroke severity between the two groups with or without elevated thyroid peroxidase antibody (TPO-Ab) and thyroglobulin autoantibody (Tg-Ab) in China.

## 2. Methods

### 2.1. Patient Selection

We followed the methods of Cho et al. [6]. We retrospectively analyzed our AIS patients with euthyroidism admitted to the First Affiliated Hospital of Tsinghua University from January 2017 to December 2018 and compared the stroke severity between these patients with or without elevated TPO-Ab and Tg-Ab. The protocols of our study had been approved by the Tsinghua University ethics committee. The inclusion criteria were AIS confirmed by magnetic resonance imaging (MRI) within 3 days of symptom onset, and the results of thyroid function, TPO-Ab, and Tg-Ab were available. Patients with an overt history of thyroid disease or diagnosed with thyroid disorder during admission; those with immunological, infectious, and toxic diseases; those undergoing thrombolytic treatment; and those on immunosuppressant drugs or immunomodulators were excluded. Thyroid function was considered based on thyroid-stimulating hormone (TSH) level. Of the initial 469 patients, 47 were excluded because of hyperthyroidism (TSH < 0.27 mU/L, *n* = 6, 1.28%) and hypothyroidism (TSH > 4.2 mU/L, *n* = 41, 8.74%). Finally, a total of 422 (TSH 0.27–4.2 mU/L, 89.98%) patients with normal thyroid function were eligible for inclusion in this study.

### 2.2. Assessment of Clinical Courses

The baseline demographics and some vascular risk factors were collected. We especially paid more attention to the effects of aspirin. Patients were considered as being on aspirin therapy if they regularly took the drug within the 3-month period before admission. The patients were diagnosed with diabetes mellitus if the fasting blood glucose was elevated (≥7.0 mmol/L) at least 2 times or if patients were on regular hypoglycemic agents. Patients were diagnosed with hypertension if their blood pressure was elevated (systolic: ≥140 mmHg or diastolic: ≥90 mmHg) or if they were on regular antihypertensive medication. The patients were diagnosed with hyperlipidemia if their fasting serum total cholesterol (≥6.2 mmol/L) or low-density lipoprotein cholesterol (≥4.1 mmol/L) was elevated, or if they were on regular lipid-lowering medication. Patients were considered smokers if they smoked at least one cigarette per day within the 3-month period before admission.

Laboratory tests included blood glucose, blood lipid profile, erythrocyte sedimentation rate, high-sensitivity C-reactive protein, fibrinogen, homocysteine, thyroid function, TPO-Ab, and Tg-Ab in consecutive patients with AIS presenting within 3 days of symptom onset. The fasting blood samples were collected in the morning after admission. The thyroid functions were evaluated by measuring the serum levels of total triiodothyronine (TT3), free triiodothyronine (FT3), total thyroxine (TT4), free thyroxine (FT4), and thyroid- stimulating hormone (TSH). The reference ranges for TT3, FT3, TT4, FT4, and TSH, respectively, were 1.3–3.1 nmol/L, 2.8–7.1 pmol/L, 66–181 nmol/L, 12–22 pmol/L, and 0.27–4.2 mU/L. For the assessment of thyroid autoimmunity, the serum concentrations of the TPO-Ab and Tg-Ab were checked using electrochemiluminescence with a commercial kit (Roche, Basel, Switzerland). We divided the AIS patients into two groups according to the levels of the thyroid autoantibodies. The positive thyroid autoantibody (PTA) group was defined as either TPO − Ab > 34 IU/mL and/or Tg − Ab > 115 IU/mL in accordance with the manufacturer's reference. The negative thyroid autoantibody (NTA) group was defined as TPO − Ab ≤ 34 IU/mL and Tg − Ab ≤ 115 IU/mL.

Stroke severity was assessed according to the National Institutes of Health Stroke Scale (NIHSS) score at admission and discharge. In addition, the mortality during hospitalizations and hospitalization time were used to evaluate the unfavorable outcome.

### 2.3. Statistical Analysis

Data were expressed as the mean ± standard deviation or percentage. Statistical analysis was performed with SPSS (version 20.0; IBM Corporation, Armonk, NY, USA) between the two groups using independent sample two-tailed *t*-test and chi-square test. *P* < 0.05 was considered to indicate statistically significant differences.

## 3. Results

Out of the included 422 patients, 50 (11.8%) were included in the PTA group. Both the TPO-Ab and Tg-Ab were elevated in 22 patients in the PTA group. There was no significant difference on the prevalence of the positive TPO-Ab and the positive Tg-Ab (*n* = 37 [8.8%] vs. *n* = 34 [8.1%], *P* = 0.509).

The baseline demographics (Table 1) and all the laboratory results including the risk factors related to the intracranial stenosis (Table 2) and the clinical features including the thyroid function (Table 3) of the PTA and NTA groups were shown in detail. Table 1 shows that more female patients had elevated thyroid autoantibodies (*n* = 25 [50.0%] vs. *n* = 113 [30.4%], *P* < 0.01) compared to the NTA group. The age and vascular risk factors were not significantly different between the two groups (*P* > 0.05). Furthermore, the rate of aspirin therapy was not significantly different between the two groups (*P* > 0.05) in Table 1. Even though the protective high-density lipoprotein cholesterol was higher in the PTA group than the NTA group (*P* < 0.01) in Table 2, the higher NIHSS scores at admission were observed in the PTA group than the NTA group (*P* < 0.05). The duration of hospital stay was similar between the two groups (*P* = 0.523). The NIHSS at discharge was evaluated again. Higher NIHSS scores at discharge could be used to predict poor prognosis and severity of AIS patients early in the PTA group than the NTA group in Table 3. Overall, seven patients died (four patients in the PTA group [8%] and three patients in the NTA group [0.8%] (*P* < 0.01)). Therefore, there was significant difference in the mortality between the PTA group and NTA group in this study.

Table 3 shows that even though the mean values of TSH, TT3, TT4, FT3, and FT4 were all within the normal reference range, the FT3 level was significantly lower in AIS patients with elevated thyroid autoantibodies (*P* < 0.05).

## 4. Discussion

This study indicated that TPO-Ab and Tg-Ab aggravate stroke severity in euthyroidism with AIS, independent of thyroid function. In our study sample, we evaluated the possible influence of thyroid autoimmunity related to elevated TPO-Ab and Tg-Ab on functional outcome in euthyroidism with AIS in China. Some previous studies have indicated that autoimmune reactions were related to the occurrence of intracranial arterial stenosis in stroke patients with hyperthyroidism [4]. Hyperthyroidism was investigated as a risk factor for vascular damages related to poor functional outcome [7]. A case report detailed the intracranial stenosis and damages noticed in Graves' disease, which were relieved after glucocorticoid treatment and plasmapheresis [8]. The above several studies have shown that abnormal thyroid function may involve short- and long-term cardiovascular and cerebrovascular bad effects. However, it is unclear whether the autoimmunity related to elevated TPO-Ab or Tg-Ab was associated with the severity of AIS. To exclude the effects of thyroid dysfunction on stroke functional outcome, we reviewed 422 patients with normal thyroid function and AIS. Some researchers have shown that male sex, diabetes, dyslipidemia, hypertension, smoking, and hyperhomocystinemia are the key risk factors of intracranial stenosis and damages in AIS patients [9, 10]. High-density lipoprotein cholesterol is one of the protective factors of atherosclerosis in ischemic stroke patients [11]. In addition, aspirin is the most commonly used antiplatelet drug and may provide greater protection against the ischemic stroke. We focused our attention on the effects of aspirin and removed the interference of aspirin therapy between the two groups. All the vascular risk factors were excluded. We found that stroke was still more serious in the PTA group, despite elevated levels of high-density lipoprotein cholesterol, which further suggested and illustrated the possible specific effects of autoimmunity related to elevated TPO-Ab or Tg-Ab on stroke severity in euthyroid state. Although the precise mechanism related to the poor functional outcome is not discussed in this study, the most likely and reasonable speculation is that the vascular damage associated with inappropriate autoimmune response may aggravate stroke severity by contributing to endothelial dysfunction and atherosclerosis [12]. The previous research reported the impairment of endothelial-dependent arterial dilatation in autoimmune thyroiditis patients with euthyroidism, indicating that thyroid autoimmunity may cause endothelial dysfunction [13]. In addition, the endothelial dysfunction was a marker of atherosclerosis risk [14]. Furthermore, brain perfusion in patients with autoimmune thyroiditis was decreased, which suggested the possible relationship between elevated thyroid autoantibodies and unfavorable brain perfusion in AIS [15]. Now that the endothelial dysfunction is recognized as an important early event in atherogenesis, further studies may be needed to clarify the relationship between the thyroid autoantibodies and endothelial dysfunction in AIS patients with normal thyroid function states.

Our study revealed more female than male patients in the group with positive thyroid autoantibodies in China, which was consistent with a previous study that indicated a clear female preponderance with a high prevalence of thyroid autoantibodies in the general population [16–19]. The ratio of female and male ranged from 1.8 : 1 to 4.4 : 1 in the above studies. Our study showed the ratio of female and male was 1 : 1 in the PTA group; however, the ratio of female and male was 1 : 2.3 in the NTA group. This significant difference between the two groups highlighted an obvious female preponderance. These monoclonal antibodies specific to estrogen receptors and progesterone receptors were present in the thyroid gland tissues [20]. Hence, there could be an association between thyroid autoimmunity reactions and sex hormone receptors. A study showed an independent association of elevated TPO-Ab with intracranial large artery damages in younger stroke patients with normal thyroid function. However, in that case, the independent relationship of elevated Tg-Ab on intracranial large artery stenosis could not be found [21]. The levels of Tg-Ab and TPO-Ab increase independently in response to thyroglobulin and thyroid peroxidase, respectively. The prevalence of positive TPO-Ab ranges from 3.4% to 13.4% in the general population. In our study, the prevalence of elevated TPO-Ab and elevated Tg-Ab was 8.8% and 8.1%, respectively. To investigate thyroid autoimmunity, we included both thyroid autoantibodies to elucidate their effects on functional outcome in patients with AIS.

Our data suggested that lower FT3 values within the normal reference range on admission in the elevated thyroid autoantibodies group may predict a poor functional outcome in AIS patients. Low T3 syndrome is known as euthyroid sick syndrome, which shows a reduction of serum T3 without the elevation of serum TSH [22]. In our study, the mean values of TSH, TT3, TT4, FT3, and FT4 were all within the euthyroid reference range. However, the lower FT3 has been described as being consistent with worse AIS severity. FT3 is generally more sensitive than TT3. The change trend of FT3 can still predict the stroke severity in AIS, even though FT3 is in the normal range.

The limitations of our study include the retrospective nature of our study and the limitation of a single academic institution. We need to further carry out large-scale prospective and multicenter studies to validate our findings.

## 5. Conclusions

Our results indicate that the elevated thyroid autoantibodies aggravate stroke severity in euthyroidism with AIS in Chinese patients. Our findings remind us that we need to further evaluate the role of the autoimmune factors in the pathogenesis of AIS, independent of thyroid function.

## Figures and Tables

**Table 1:** Comparison of demographic features in AIS and euthyroidism with and without elevated thyroid autoantibodies.

	PTA (*n* = 50)	NTA (*n* = 372)	*P* value
Age, years	72.8 ± 10.9	76.4 ± 14.0	0.518
Female, *n* (%)	25 (50.0)	113 (30.4)	*P* < 0.01^∗∗^
Hypertension, *n* (%)	46 (92.0)	332 (89.2)	0.550
Diabetes, *n* (%)	29 (58.0)	197 (53.0)	0.503
Hyperlipidemia, *n* (%)	29 (58.0)	234 (62.9)	0.502
Cigarette smoking, *n* (%)	11 (22.0)	115 (30.9)	0.192
Aspirin therapy, *n* (%)	11 (22.0)	97 (26.1)	0.529

Data presented as mean ± standard deviation or percentage. Abbreviations: AIS: acute ischemic stroke; PTA: positive thyroid autoantibody; NTA: negative thyroid autoantibody. ^∗^*P* < 0.05 and ^∗∗^*P* < 0.01.

**Table 2:** Comparison of the risk factors related to the intracranial stenosis in AIS and euthyroidism with and without elevated thyroid autoantibodies.

	PTA (*n* = 50)	NTA (*n* = 372)	*P* value
Blood pressure at admission			
Systolic blood pressure (mmHg)	151.4 ± 19.2	149.2 ± 21.5	0.482
Diastolic blood pressure (mmHg)	86.7 ± 12.6	85.0 ± 13.8	0.408
Blood lipid profile			
Total cholesterol (mmol/L)	4.57 ± 1.06	4.31 ± 1.07	0.118
Triglycerides (mmol/L)	1.47 ± 1.1	1.54 ± 1.02	0.626
High-density lipoprotein cholesterol (mmol/L)	1.33 ± 0.41	1.18 ± 0.32	*P* < 0.01^∗∗^
Low-density lipoprotein cholesterol (mmol/L)	3.0 ± 1.02	2.9 ± 1.27	0.571
Blood glucose profile			
Fasting plasma glucose (mmol/L)	7.21 ± 3.44	6.78 ± 2.6	0.296
Glycosylated hemoglobin (%)	7.0 ± 2.3	6.6 ± 1.6	0.238
Erythrocyte sedimentation rate (mm/h)	10.6 ± 8.4	8.7 ± 11.9	0.33
High-sensitivity C-reactive protein (mg/L)	10.31 ± 17.8	9.97 ± 21.71	0.92
Fibrinogen (g/L)	3.18 ± 0.77	3.23 ± 0.81	0.685
Homocysteine (umol/L)	16.26 ± 12.67	17.33 ± 14.08	0.618

Data presented as mean ± standard deviation or percentage. Abbreviations: AIS: acute ischemic stroke; PTA: positive thyroid autoantibody; NTA: negative thyroid autoantibody. ^∗^*P* < 0.05 and ^∗∗^*P* < 0.01.

**Table 3:** Comparison of thyroid function and clinical features in AIS and euthyroidism with and without elevated thyroid autoantibodies.

	PTA (*n* = 50)	NTA (*n* = 372)	*P* value
Thyroid function test			
TT3 (pmol/L)	1.67 ± 0.33	1.62 ± 0.35	0.350
FT3 (pmol/L)	3.83 ± 0.54	4.02 ± 0.74	0.025^∗^
TT4 (pmol/L)	99.86 ± 17.14	96.39 ± 21.94	0.288
FT4 (pmol/L)	16.33 ± 2.6	16.54 ± 4.71	0.753
TSH (mU/L)	1.76 ± 1.17	1.68 ± 0.92	0.632
NIHSS score at admission	5.3 ± 5.4	3.9 ± 3.9	0.025^∗^
NIHSS score at discharge	3.9 ± 4.2	2.8 ± 3.1	0.023^∗^
Mortality, *n* (%)	4 (8%)	3 (0.8)	*P* < 0.01^∗∗^
Duration of hospital stay (days)	14.3 ± 7.1	14.8 ± 7.1	0.523

Data presented as mean ± standard deviation or percentage. Abbreviations: AIS: acute ischemic stroke; PTA: positive thyroid autoantibody; NTA: negative thyroid autoantibody; TT3: total triiodothyronine; FT3: free triiodothyronine; TT4: total thyroxine; FT4: free thyroxine; TSH: thyroid-stimulating hormone; NIHSS: National Institutes of Health Stroke Scale score. ^∗^*P* < 0.05 and ^∗∗^*P* < 0.01.

## Data Availability

The data used to support the findings of this study are included within the article.
